# Corticosteroids, serum proteins, and the next phase of precision medicine in IgA nephropathy

**DOI:** 10.1093/ckj/sfag345

**Published:** 2026-10-05

**Authors:** Carmine Zoccali, Jürgen Floege

**Affiliations:** Istituto di Biologia e Genetica Molecolare (BIOGEM), Ariano Irpino, Italy; Associazione Ipertensione Nefrologia e Trapianto Renale (IPNET), Reggio Calabria, Italy; Division of Nephrology, University Hospital, Rheinisch-Westfälische Technische Hochschule (RWTH) Aachen, Aachen, Germany; Istituto di Biologia e Genetica Molecolare (BIOGEM), Ariano Irpino, Italy; Division of Nephrology, University Hospital, Rheinisch-Westfälische Technische Hochschule (RWTH) Aachen, Aachen, Germany

**Keywords:** corticosteroids, IgA, genetics, proteomic

## Abstract

Systemic glucocorticoids remain an important but problematic treatment option in high-risk IgA nephropathy, offering kidney protection at the cost of clinically relevant toxicity. Recent serum proteomic analyses embedded within the randomized TESTING trial provide a new framework for identifying patients most likely to benefit from methylprednisolone. In 241 Chinese participants with IgA nephropathy, longitudinal profiling of 479 serum samples showed that methylprednisolone significantly modulated 302 proteins involved in cytoskeletal regulation, extracellular matrix interactions, immunometabolism, coagulation, protein turnover, and related pathways. Importantly, complement- and endothelial injury-associated pathways were incompletely modified and remained linked to adverse kidney outcomes, suggesting residual disease mechanisms that may require complementary targeted therapies. Among baseline proteins, leptin and C1QTNF5 emerged as practical candidate biomarkers of glucocorticoid responsiveness, improving prediction of treatment efficacy beyond conventional clinical variables. These findings support a shift from purely prognostic risk stratification toward treatment-informed precision medicine in IgA nephropathy. However, the biomarker model requires prospective validation across diverse populations, steroid regimens, and background therapies, and future tools must integrate both predicted kidney benefit and treatment-related harm. If confirmed, serum proteomics could help clinicians move beyond population-average steroid decisions toward biologically guided selection of patients most likely to benefit while avoiding unnecessary toxicity.

For decades, IgA nephropathy (IgAN) has occupied an uncomfortable space between clinical familiarity and biological uncertainty. It is the most common primary glomerulonephritis worldwide, a major cause of kidney failure in young adults, and yet it was one of the field’s most difficult therapeutic puzzles. The disease begins with disordered mucosal immunity and pathogenic IgA-containing immune complexes, but its clinical course is shaped by a broader cascade of glomerular inflammation, complement activation, endothelial injury, tubulointerstitial fibrosis, and progressive nephron loss. The result is a syndrome in which the same diagnostic label can encompass strikingly different trajectories and treatment responses.

Systemic glucocorticoids have long been both indispensable and problematic in this landscape. The TESTING trial [1] provided the most persuasive randomized evidence that oral methylprednisolone can reduce kidney disease progression in high-risk Asian patients with IgAN, but it also exposed the cost of this benefit: serious, sometimes even lethal infections and other steroid-related toxicities, particularly with the higher-dose regime. Subsequently, a modified lower-dose strategy, combined with infection prophylaxis, preserved much of the renal benefit with improved safety, but even here one infection-related death occurred with corticosteroids. TESTING thus helped to restore a carefully circumscribed role for systemic steroids in contemporary IgAN care. Yet the central clinical question remains unresolved: Who should receive glucocorticoids and who should be spared them?

A recent study by Wang *et al*. [2] addressed this question with an unusually powerful design. Rather than conducting biomarker discovery in an observational cohort, the investigators embedded serum proteomic profiling within the randomized, placebo-controlled TESTING trial. They analyzed 479 longitudinal serum samples from 241 Chinese participants with IgAN, collected at baseline and at 6 and 12 months, and quantified >1500 proteins by data-independent acquisition mass spectrometry. By exploiting randomization, longitudinal sampling, and placebo comparison, the authors could distinguish proteins modulated by methylprednisolone from those merely associated with disease activity or progression. This distinction is not semantic (Fig. 1). It is the difference between biomarkers of prognosis and biomarkers of treatment biology. Unlike recent efforts to estimate individual corticosteroid benefit from clinical [3] and histologic variables [4], Wang *et al*. [2] add a treatment-engaged molecular layer by asking which circulating proteins methylprednisolone actually modulates within a randomized trial.

**Figure 1:**
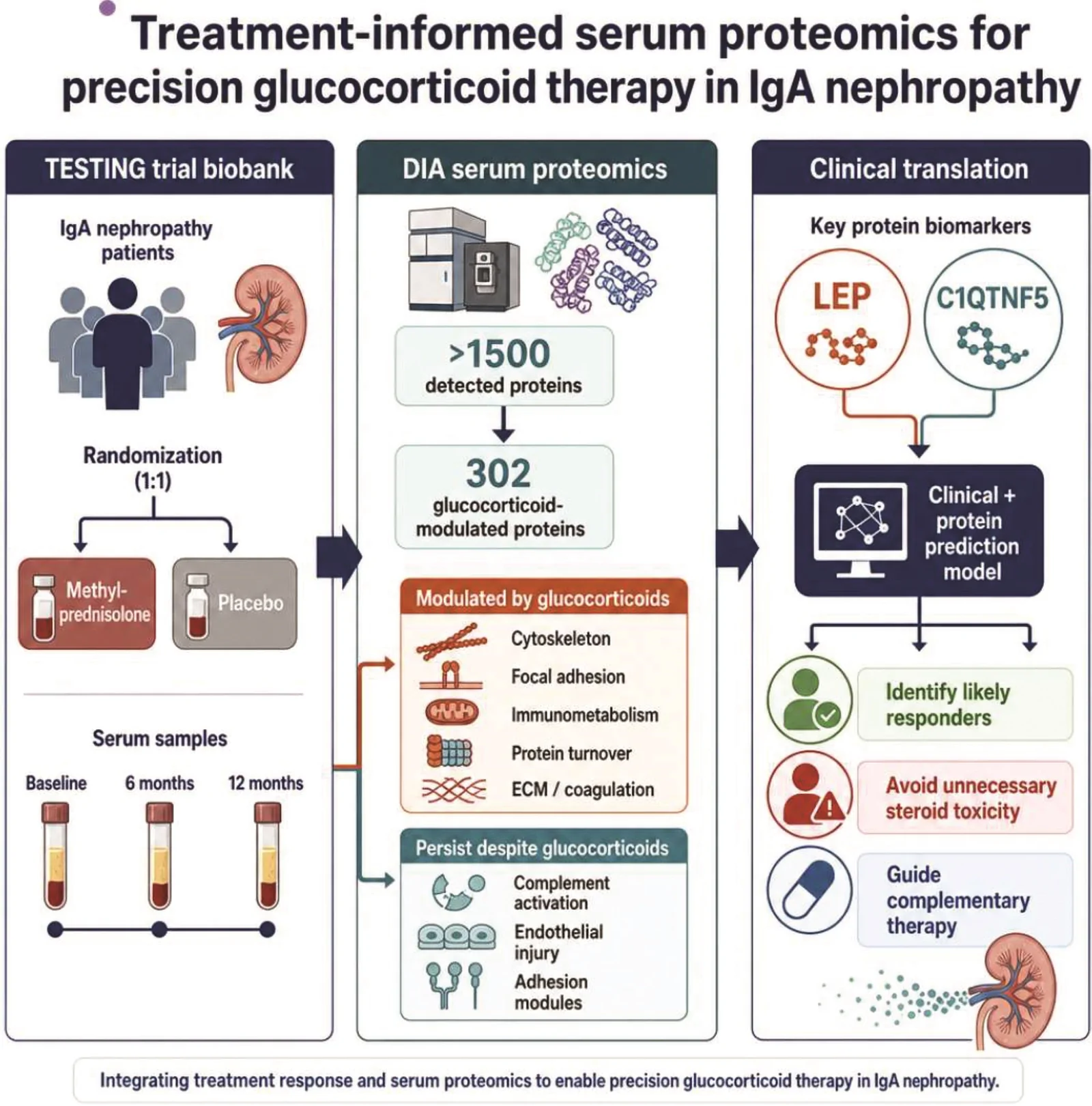
Treatment-informed serum proteomics for precision glucocorticoid therapy in IgAN. Longitudinal serum proteomics embedded within the randomized TESTING trial separates glucocorticoid-modulated pathways from residual disease-associated pathways. Baseline proteins, particularly Leptin (LEP) and C1QTNF5, improve prediction of methylprednisolone benefit when added to clinical variables and may help identify likely responders, avoid unnecessary steroid toxicity, and guide complementary targeted therapy.

The headline finding is that 302 serum proteins were significantly modulated by methylprednisolone over 12 months. These proteins were enriched in pathways related to cytoskeletal regulation, focal adhesion, extracellular matrix–receptor interaction, protein turnover, immunometabolism, coagulation, and complement/coagulation cascades. Several observations are biologically plausible and clinically intriguing. Glucocorticoids appeared to influence proteins involved in actin cytoskeletal stability and cell–matrix interactions, consistent with experimental evidence that steroids can stabilize podocyte actin architecture [5, 6]. They also altered ribosomal, mitochondrial ribosomal, proteasomal, metabolic, and coagulation-related proteins, reinforcing the concept that glucocorticoid action in kidney disease extends way beyond generic immunosuppression.

Equally important is what methylprednisolone did not substantially alter. Complement- and endothelial injury-associated modules, including networks centered on proteins such as C3, complement factor H, complement factor B, Neural Cell Adhesion Molecule 1 (NCAM1), syndecan-1, and l-selectin, remained incompletely modulated despite treatment but correlated with adverse kidney outcome. This negative space in the proteomic map suggests that glucocorticoids, even when effective, leave important disease circuits untouched. Interestingly, targeted-release budesonide (nefecon) has been shown to modulate circulating proteins of the alternative and mannose-binding lectin pathways of complement [7], suggesting that possibly not all corticosteroids work via similar mechanisms. In addition, knowledge of unaltered pathways may provide the basis for future rational combination or sequencing therapies picking from the many emerging new therapeutic options in IgAN [8].

Among glucocorticoid-modulated proteins, the investigators [2] identified 20 baseline protein levels that modified the effect of methylprednisolone versus placebo on the primary kidney outcome. Further modeling prioritized a smaller set, with leptin, C1q, and tumor necrosis factor-related protein 5 (C1QTNF5), Kinase Insert Domain Receptor (KDR), Proteasome 20S Subunit Beta 8 (PSMB8), nuclear pore glycoprotein-210 (NUP210), Ribosomal Protein Large 8 (RPL8), and others contributing to prediction. Ultimately, ELISA validation supported leptin and C1QTNF5 as practical candidate biomarkers. A model incorporating these proteins improved prediction of glucocorticoid efficacy beyond clinical variables alone, with a reported 5-year area under the curve (AUC) of 0.85 compared with 0.80 for the clinical model.

This is a potentially important advance because most existing approaches to treatment selection in IgAN rely on proteinuria, estimated glomerular filtration rate, blood pressure, histology, and sometimes genetic information. These variables are clinically useful, but they are not drug-specific. Proteinuria, for example, is both a risk marker and a therapeutic target, but it does not reveal whether a patient’s disease biology is likely to be steroid-responsive. A serum proteomic signature derived from randomized treatment response moves closer to the ideal of predictive, not merely prognostic, medicine. Beyond protein analyses, genetic variants are stable and mechanistically informative, but they do not measure the dynamic state of immune-metabolic activation. Histological lesions capture accumulated injury and current inflammatory activity but cannot fully reflect systemic treatment-engaged pathways. However, as with protein analyses, more sophisticated analyses may improve the prognostic power of histology and we have recently published a number of novel tissue parameters derived from morphometric data that were powerful predictors of corticosteroid benefits or lack thereof in individuals with IgAN [9]. Whether a combined proteomic and pathomic signature can further improve the prediction of steroid-responsiveness remains to be proven.

In the study of Wang *et al*. [2], serum leptin emerged as an especially interesting signal. Traditionally viewed as an adipokine regulating appetite and energy homeostasis, leptin is now recognized as a mediator linking metabolism and immunity. It can promote Th1 and Th17 responses, restrain regulatory T-cell activity, and influence inflammatory tone [10]. In the kidney, leptin has been implicated in glomerular transforming growth factor-beta signaling, extracellular matrix accumulation, mesangial activation, and glomerulosclerosis. In the present study, higher baseline leptin identified patients deriving greater methylprednisolone benefit, and leptin levels declined after treatment. One interpretation is that elevated leptin marks a steroid-modifiable immunometabolic inflammatory state. If so, leptin may be less a passive biomarker than a window into a tractable disease phenotype.

C1QTNF5 is less familiar to nephrologists but no less intriguing. As a member of the C1q/TNF-related protein family, it has been linked to metabolic regulation, vascular biology, inflammation, adiponectin signaling, and extracellular matrix biology [11]. In this study, lower baseline C1QTNF5 identified patients with greater methylprednisolone benefit, whereas higher levels appeared to mark reduced efficacy. The biological explanation remains uncertain, but the finding points toward a broader immunometabolic and tissue-remodeling axis in IgAN. Importantly, both leptin and C1QTNF5 are quantifiable by ELISA, making eventual clinical translation more feasible than complex mass-spectrometry-only panels.

Nevertheless, important limitations of the above proteomic approach remain. First, the study is nested within the Chinese cohort of TESTING, and external validation across different ancestry groups, healthcare settings, steroid species and regimens, and background therapies will be required. IgAN is heterogeneous across geography and ethnicity [12, 13]. A biomarker model that performs well in one population may recalibrate poorly in another.

Second, although the randomized design strengthens causal inference about treatment-modulated protein trajectories, the prediction model itself still faces the familiar risks of biomarker overfitting. [14] The number of clinical events, the dimensionality of proteomic data, the selection of interaction terms, and the internal train-test split all matter. Ridge penalization, an established technique to limit overfitting used by Wang *et al*., is appropriate [15], but does not replace prospective validation [16]. The reported AUC improvement is promising, but clinical utility will depend on calibration, decision-curve analysis, net benefit, threshold effects, assay reproducibility, and whether the model changes treatment decisions in ways that improve outcomes.

Third, the outcome predicted in Wang’s study is kidney benefit, not net clinical benefit. This distinction is crucial for glucocorticoids. A patient predicted to derive renal benefit may still face unacceptable infection, metabolic, psychiatric, skeletal, or cardiovascular risks. Conversely, a patient with modest predicted renal benefit but very low adverse-event risk might reasonably choose therapy. As in oncology research [17], future decision tools should integrate both efficacy and toxicity prediction, especially because the therapeutic dilemma in IgAN is not whether steroids work in Asian patients, but whether their benefit justifies their risk in a given individual.

Fourth, the biomarker findings should not be read as ready for routine clinical use. At present, neither leptin nor C1QTNF5 should be used to decide on glucocorticoid therapy outside research or prospective validation frameworks. Assay standardization, reference ranges, pre-analytical stability, biological variability, and cut points require careful development. The median-based dichotomization used in the study is analytically convenient but may not correspond to clinically portable thresholds.

The broader importance of this work lies in its methodological template. Randomized trials are rarely used to their full biological potential. They are usually designed to answer whether an intervention works, not why it works, in whom it works, and what pathways remain active despite therapy. Longitudinal omics embedded in randomized trials can answer these questions more convincingly than observational biomarker studies. In IgAN, where therapeutic options are multiplying rapidly, this approach could help classify patients according to dominant actionable biology: mucosal immune activation, complement dysregulation, podocyte–cytoskeletal vulnerability, endothelial injury, metabolic inflammation, or fibrotic progression. Such stratification is urgently needed. The field is moving from a binary world of supportive care versus systemic steroids to a crowded therapeutic ecosystem [18]. Without predictive biomarkers, clinicians will be left to sequence expensive and potentially toxic therapies by trial and error. With validated treatment-informed biomarkers, IgAN could become a model for precision nephrology.

Wang and colleagues [2] have therefore provided more than a candidate two-protein classifier. They have shown that the biology of treatment response can be mapped in human randomized trials; that glucocorticoids reshape cytoskeletal, immune-metabolic, coagulation, and extracellular matrix pathways; that complement and endothelial injury may persist as residual targets; and that serum proteins can improve prediction of steroid benefit beyond conventional clinical variables. The discovery is not yet practice changing, but it is direction changing.

The next steps are clear. The leptin and C1QTNF5 signals should be validated prospectively in ethnically diverse cohorts and ideally in randomized datasets. Models should be tested against clinically meaningful decision thresholds, not only discrimination metrics. Biomarker-guided treatment algorithms should include adverse-event prediction. Finally, mechanistic experiments should interrogate whether leptin and C1QTNF5 are merely markers of steroid responsiveness or participants in the causal pathways that determine it.

Glucocorticoids have always been blunt instruments. This study suggests that their use in IgAN need not remain blunt. If confirmed, serum proteomics may allow clinicians to replace a population-average decision with a biologically informed one: not simply “Should steroids be used in IgAN?” but “‘Is this patient’s disease biology likely to respond to glucocorticoids, and what pathways require another strategy?” That is the promise of precision nephrology, and this work brings it appreciably closer.
